# An Indeterminate Choroidal Melanocytic Lesion: A Case Report

**DOI:** 10.7759/cureus.96313

**Published:** 2025-11-07

**Authors:** Salah Alrashidi, Mariam Alenezi

**Affiliations:** 1 Ophthalmology, Farwaniya and Kuwait Sidra Hospital, Kuwait City, KWT

**Keywords:** choroid, choroidal melanoma, choroidal nevi, indeterminate choroidal melanocytic lesion, pigmented choroidal lesions

## Abstract

Choroidal nevi are common, benign growths in the eye that are usually harmless but sometimes can present with atypical features, making them challenging to diagnose and distinguish from early-stage melanoma.

In this case report, we discuss the case of a 52-year-old male patient with a history of intermittent dryness, blurriness, and floaters in his right eye. Presented to ophthalmology, incidentally revealing a suspected choroidal mass, and we document his subsequent monitoring over a four-year period. Despite the patient developing some risk factors for malignant transformation, such as subretinal fluid, the lesion thickness decreased. Interestingly, the patient’s history of gastric sleeve surgery led to a decrease in vitamin B12 levels. This may have played a role in the lesion’s behavior, although no direct link to malignancy progression has been established in the literature.

This case demonstrates the importance of individualized care, highlighting that not all lesions with worrisome features evolve into melanoma. Close observation and long-term monitoring can be considered an effective strategy, especially in patients without risk factors.

This case challenges the standard thinking on choroidal nevi and reinforces the need for personalized management plans, particularly in patients with unusual presentations.

## Introduction

Choroidal nevi are benign melanocytic tumors of the posterior uveal tract and are relatively common findings in routine ophthalmic examination. Most choroidal nevi are asymptomatic and exhibit characteristic features such as flat or minimally elevated pigmented lesions with well-defined margins, typically less than 2 mm in thickness [1]. However, some nevi may present with atypical features or exhibit growth over time, raising concern for transformation into malignant melanoma, a potentially life- and vision-threatening condition. Distinguishing between a benign nevus and an early-stage melanoma can be clinically challenging, especially in lesions with borderline features. These lesions are often classified as ‘indeterminate choroidal melanocytic lesions’ and require close observation, multimodal imaging, and follow-up to guide appropriate management. 

## Case presentation

A 52-year-old male patient initially presented to ophthalmology causality at the age of 48 years old with complaints of intermittent dryness and blurriness in the right eye, associated with a long history of floaters. He denied experiencing flashes, periocular pain, diplopia, or any history of ocular trauma.

His medical history was significant for bronchial asthma, hypertension, and a gastric sleeve surgery performed 10 years ago. Family history was notable for ocular malignancy in his niece, who was diagnosed at the age of four.

**Figure 1:**
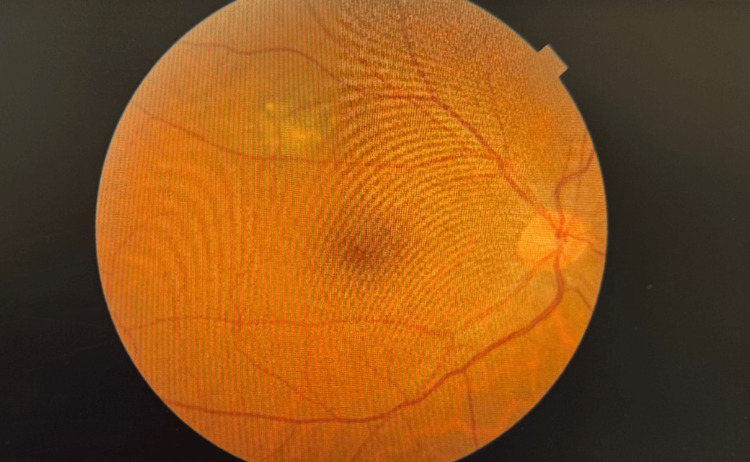
Fundus photography of the right eye

Initial examination for both eyes done in 2021 had the following results: best corrected visual acuity (BCVA) was 20/20. The anterior segment had normal conjunctiva, a clear cornea, and a deep and quite anterior chamber. Intraocular pressure was 18 mmHg (within normal limits), and extraocular movements were full and unrestricted. Fundus examination was unremarkable in the left eye but revealed a suspected supratemporal choroidal mass in the right eye, as shown in Figure 1.

**Figure 2:**
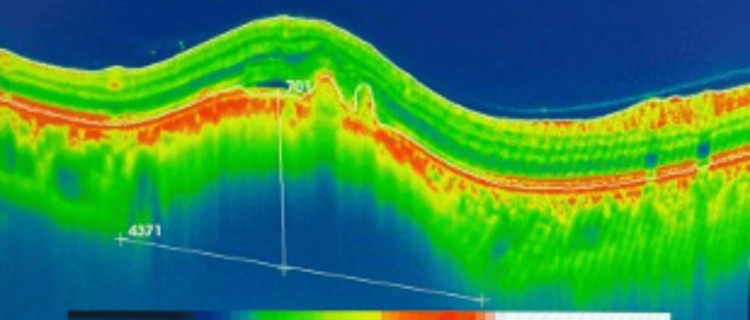
Optical coherence tomography of the indeterminate choroidal lesion of right eye in the previous visit

**Figure 3:**
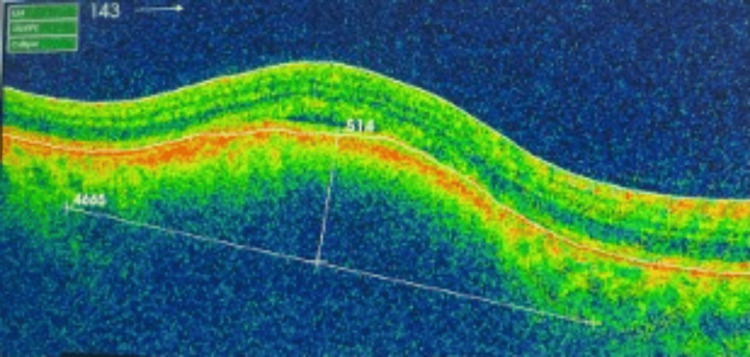
Optical coherence tomography of the indeterminate choroidal lesion of right eye in the latest visit (April 2025)

The patient was followed up regularly and closely in the ocular oncology clinic. At the most recent follow-up in April 2025 (four years after initial presentation), his BCVA remained 20/20 in both eyes. The left eye continued to show no abnormalities; however, the examination of the right eye revealed a mild posterior subcapsular cataract in the anterior segment. In the posterior segment, the optic disc appeared normal, and the macula was dry, though minimal subretinal fluid was noted, which was contributing to mild metamorphopsia. Importantly, the previously noted choroidal lesion had decreased in thickness from 701 µm (Figure 2) to 514 µm at this visit in April 2025 (Figure 3).

## Discussion

This case of an indeterminate choroidal melanocytic lesion presents several key features that differ from typical patterns observed in the literature. While choroidal nevi are generally benign, their potential for malignant transformation remains a significant concern. Epidemiological studies have linked factors such as female sex and Caucasian ethnicity to a higher prevalence of nevi and thus progression into melanoma [1]. However, our patient deviates from these trends, as he is a male and Middle Eastern, highlighting that such lesions can develop regardless of the expected demographic profiles.

In the literature, no correlation has yet been established between progression or regression of ocular nevi or malignancies with vitamin deficiencies. However, a correlation has been found between vitamin D levels and choroidal thickness [2]. Additionally, some epidemiological studies suggested that high vitamin D levels may be protective against malignant cutaneous melanoma. However, there are no clear indications to recommend vitamin D supplementation [3]. Interestingly, a nonrandomized controlled trial by Taube et al. titled “Association of Bariatric Surgery with Skin Cancer Incidence in Adults with Obesity” found a reduction in the incidence of skin cancers, including cutaneous malignant melanoma; this study suggests an association between obesity and skin cancer formation [4].

Our patient experienced a decrease in vitamin B12 following gastric sleeve surgery, coinciding with the development of subretinal fluid and a reduction in lesion thickness. This unique combination of factors raises questions about the role of nutrition and metabolic changes in the lesion’s behavior.

One of the key risk factors of malignant transformation in choroidal melanocytic lesions is the presence of subretinal fluid, which is often associated with increased lesion activity and a higher risk of progression to melanoma [5]. However, in our case, despite the appearance of minimal subretinal fluid during the last follow-up, the lesion’s thickness gradually decreased, suggesting a more benign or regressive course rather than malignant transformation. This finding emphasises the need for careful, individualized follow-up.

Indeterminate choroidal melanocytic lesions occupy a critical space in the management of ambiguous presentations. These lesions often fall between benign nevi and early melanomas, making them difficult to categorize. A cautious, multimodal approach is essential, incorporating tools like the “To Find Small Ocular Melanoma Using Helpful Hints Daily” mnemonic, risk stratification, and long-term monitoring [5]. In this case, the patient maintained stable visual acuity over a four-year period, with gradual regression of the lesion, supporting a conservative management approach.

In summary, this case reinforces the clinical variability of choroidal melanocytic lesions and highlights the importance of personalized surveillance and treatment strategies, rather than relying solely on population-level risk factors.

## Conclusions

This case highlights the importance of individualized assessment and follow-up in managing indeterminate choroidal melanocytic lesions. Despite the presence of subretinal fluid, a known risk factor for malignant transformation, the lesion regressed over time, and the patient remained asymptomatic with stable vision. It reinforces that not all lesions with concerning features follow a malignant course and that careful observation can be a safe and effective management strategy in selected cases. 
